# Dual-Wavelength Lasing Due to Second Phase Inclusions in MAPbCl_3_

**DOI:** 10.1021/acs.jpcc.5c05466

**Published:** 2025-11-12

**Authors:** Christina Siaitanidou, Violeta Spanou, Nikolaos G. Chatzarakis, Panagiotis Oikonomopoulos, Giannis S. Papaefstathiou, Katerina Tsagaraki, Eric Delamadeleine, Constantinos C. Stoumpos, Nikolaos T. Pelekanos

**Affiliations:** † Department of Materials Science & Engineering, 37777 University of Crete, P.O. Box 2208, 70013 Heraklion, Greece; ‡ Microelectronics Research Group, IESL-FORTH, 70013 Heraklion, Greece; § Department of Chemistry, University of Crete, P.O. Box 2208, 70013 Heraklion, Greece; ∥ Laboratory of Inorganic Chemistry, Department of Chemistry, 68993 National and Kapodistrian University of Athens, 15771 Athens, Greece; ⊥ Univ. Grenoble-Alpes, CEA, Grenoble INP, IRIG, PHELIQS, 38000 Grenoble, France; # Photonics of Crystals Laboratory, Saint Petersburg State University, Ulyanovskaya d.1, Saint Petersburg 198504, Russia

## Abstract

We report for the first time dual-wavelength lasing at 78 K in a vertical cavity containing ultrasmooth MAPbCl_3_ single crystals. To understand this unusual lasing behavior, the MAPbCl_3_ single crystals were thoroughly investigated in terms of temperature-dependent optical experiments. Microreflectivity measurements reveal, besides the standard exciton feature at the ∼385 nm orthorhombic bandgap, an additional previously unreported excitonic peak at ∼412 nm. This second exciton feature aligns with the second lasing line, strongly suggesting the coexistence of a “second phase” within the primary orthorhombic lattice. We show that this second phase persists up to 300 K and is likely associated with the quantum dot-like nanostructures detected on the surface of the MAPbCl_3_ crystals. The second phase depends on the growth method and crystal size but is likely traceable in all MAPbCl_3_ systems. These insights offer enhanced understanding of the MAPbCl_3_ system and open new pathways for blue-UV photonic devices.

## Graphical abstract

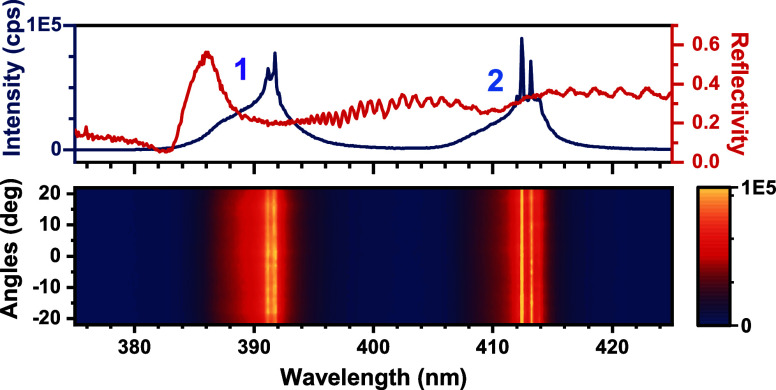

## 1 Introduction

Lead halide perovskites of the type APbX_3_, where X is a halogen atom and Α is an organic or inorganic cation such as methylammonium or Cs^+^, have attracted wide interest in the past decade, based on their remarkable optoelectronic properties^1^ and outstanding achievements in the field of photovoltaics.^2–4^ Compared to the standard technological semiconductors, e.g. silicon or GaAs, perovskites dispose of certain crucial advantages such as solution processability and low-cost fabrication, inciting further research on other optoelectronic applications beyond photovoltaics, such as photodetectors,^5,6^ γ-ray detectors,^7^ light-emitting diodes^8,9^ and lasers.^10^ Up to now, the vast majority of works in the field involve iodine-based perovskites, with an energy gap in the near-infrared which is well suited for solar cell applications. By contrast, relatively few are the works dealing with the chloride-based perovskites, having an energy gap in the deep blue-near-ultraviolet (UV) part of the spectrum.^11–16^

In this work, we study the lasing process in a vertical-cavity surface-emitting laser (VCSEL) structure, using as active medium high quality single crystals of methylammonium (MA) lead chloride (CH_3_NH_3_PbCl_3_ or MAPbCl_3_) in between Distributed Bragg Reflectors (DBRs). MAPbCl_3_ has an energy gap at ≈385 nm at low temperatures, where the single crystal takes an orthorhombic crystal structure, and at 405 nm at room temperature, where the crystal structure becomes cubic. The crystal phase transition from cubic to orthorhombic occurs around 170 K, passing through an intermediate tetragonal phase during a narrow temperature range of ∼10 degrees.^17^ An optically pumped MAPbCl_3_ VCSEL has been realized before by Tian et al.,^18^ exhibiting a deep-blue lasing emission in the 414–435 nm range, with a threshold of ∼200 μJ/cm^2^. Moreover, Yang et al. demonstrated lasing emission at ≈395 nm from a single crystal in the orthorhombic phase under multiphoton excitation.^19^ Nevertheless, none of these works analyzes the observed lasing characteristics in terms of the excitonic levels in the system, missing important information that we attempt to unveil here.

Specifically, we demonstrate for the first-time dual wavelength lasing, occurring at 414 and 391 nm (≈176 meV apart) at different thresholds in a MAPbCl_3_-containing laser cavity at 78 K. Dual-wavelength lasers can find applications in various fields such as in wavelength-division multiplexing,^20^ optical sensors,^21^ biomedical research^22,23^ and precision spectroscopy.^24^ To understand the complex optical behavior of our peculiar system, the MAPbCl_3_ lasing structures are extensively studied in terms of photoluminescence (PL) and reflectivity (RFL) as a function of temperature, based on which the dual wavelength lasing is attributed to inclusions of some “second phase” inside the orthorhombic lattice at low temperatures. We show that this second phase persists up to room temperature, likely depends on the growth method and crystal size, and is possibly a more general feature of the MAPbCl_3_ system than initially supposed. Moreover, it is quite probable that the second phase is related to the quantum dot-like nanostructures observed on the surface of the MAPbCl_3_ crystals by atomic force microscopy (AFM). Our findings offer a new perspective on the MAPbCl_3_ system and may lead to novel optoelectronic applications in the near UV.

## 2 Experimental Methods

For the fabrication of MAPbCl_3_ single crystals inside a vertical cavity, the space-confined method between DBRs was employed, as in previous works.^18,25^ For that purpose, DBRs, consisting of 8 pairs of SiO_2_/Ta_2_O_5_ on Al_2_O_3_ substrates, were designed and fabricated by electron-gun evaporation to operate in the deep blue-near UV region where MAPbCl_3_ single crystals emit. Figure 1a shows the absolute reflectivity spectrum of a DBR, normalized using an aluminum mirror, having a 98% reflectivity in a spectral window covering the emission of MAPbCl_3_ single crystals in the whole range of temperatures. The use of the Al_2_O_3_ substrate ensures transparency in the UV, upon excitation from the top of the cavity. To grow single crystals between DBRs, a solution of MAPbCl_3_ was prepared using a 0.7 M solution of DMF/DMSO (1:1) of CH_3_NH_3_Cl and PbCl_2_ in stoichiometric ratio (see Materials and Methods in Supporting Information). Next, DBRs were placed on a Petri-dish and subjected to O_2_ plasma treatment to activate the DBR surfaces and favor the crystallization of the perovskite single crystals. A typical amount of 5 μL of the above solution was drop-casted on a bottom DBR with area of ∼1 cm^2^. Then, as schematically depicted in Figure 1b, another piece of the same DBR was placed on top, face-down, forming a “sandwich” with the solution encapsulated. By applying some pressure, the solution was uniformly spread, filling the air gap between them. Finally, the samples were placed in a preheated oven set at 60 °C for 24 h, where the crystallization took place, thus forming MAPbCl_3_ single crystals in the form of thin films, with lateral dimensions of several hundred micrometers and thicknesses of 2–10 μm, as measured by scanning electron microscopy (SEM). Such thicknesses are large for VCSEL operation and explain the lateral lasing phenomena discussed below. Figure 1c shows an optical microscope image of a single crystal grown inside the cavity. The MAPbCl_3_ single-crystalline thin film exhibits a large, smooth surface with well-defined sharp edges and appears to be in homogeneous contact with both DBRs, without any air gap formation, thereby favoring the demonstration of lasing. This observation is further supported by SEM images (c.f. Figure 1d), where a uniform flat surface is observed over most of the crystal’s area. In fact, as will be shown later by AFM measurements, the crystal surface is atomically flat over extended areas, larger than 10 μm × 10 μm.

**Figure 1:**
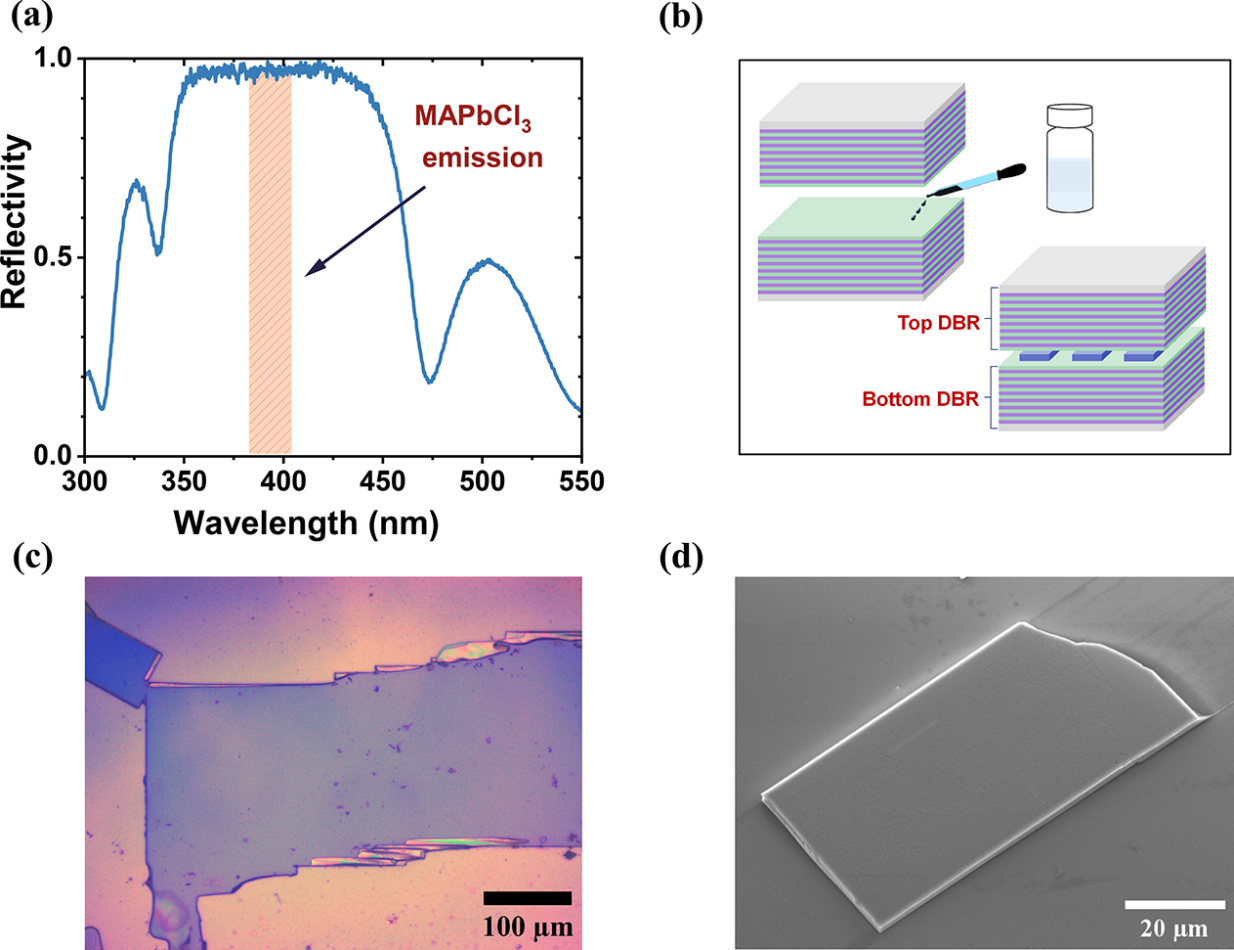
(a) Normalized Reflectivity of the actual DBRs used in the cavities. (b) Schematic illustration of solution processed MAPbCl_3_ cavity (c) Optical microscope image of a space-confined MAPbCl_3_ single crystal grown in a vertical cavity. (d) Scanning electron microscope image of a space-confined MAPbCl_3_ single crystal sitting on a bottom DBR after having removed the top DBR.

## 3 Results and Discussion

The next step was to characterize the cavity samples by microphotoluminescence (μ-PL) measurements. For that purpose, a pulsed 266 nm laser with 7.58 kHz repetition rate and 0.51 ns pulsewidth, focused down to a 60 × 10 μm^2^ spot, was used as excitation source in order to optically pump the perovskite VCSEL. For details of optical characterization setup refer to Supporting Information. The pumping spot was typically in the plane and away from the edges of the single crystals, ruling out the possibility for whispering-gallery mode type of lasing. We performed power-dependent measurements to record the emission spectra below and above lasing threshold at 78 K, as depicted in Figure 2a. Below lasing threshold, we observe two spontaneous emission lobes, the first one centered at 388 nm and the second at 412 nm. The first lobe has a visible triplet-like structure and is atributted to near-gap emission of the orthorhombic phase of MAPbCl_3_, whereas the second lobe was initially assigned to emission at defect-related states, typically present in MAPbCl_3_ single crystals in this energy range.^19,26–28^ Somewhat unexpectedly, however, with increasing the optical pump power, we observe that laser-like emission appears first on the second lobe at 414 nm, with an average power density threshold of 8 W/cm^2^, while the corresponding threshold energy per pulse is 1.05 mJ/cm^2^. As shown in Figure 2b, aside from the characteristic intensity nonlinearity, the lasing threshold is accompanied by a pronounced spectral narrowing, from about 50 meV full width at half-maximum (fwhm) below threshold to about 1 meV above threshold, further confirming the onset of lasing at 414 nm. By increasing further the power density, we notice that when the 414 nm laser line begins to saturate (c.f. Figure 2b), another sharp laser line appears simultaneously at 391 nm, which corresponds to the lower energy side of the first lobe, with a power threshold of ∼24 W/cm^2^ (threshold energy per pulse ∼3.15 mJ/cm^2^). The 391 nm line also exhibits a drastic line narrowing at threshold, further substantiating its assignment as a laser line. Similar dual-wavelength lasing has been observed on several MAPbCl_3_ VCSEL structures, an example of which is shown in Figure S1a,b of the Supporting Information. Based on the above, we ought to reconsider our initial assertion of the low-energy lobe being a defect-related state, considering that lasing at a defect level is highly unlikely in bulk semiconductors, due to the lack of sufficient density of states.

**Figure 2:**
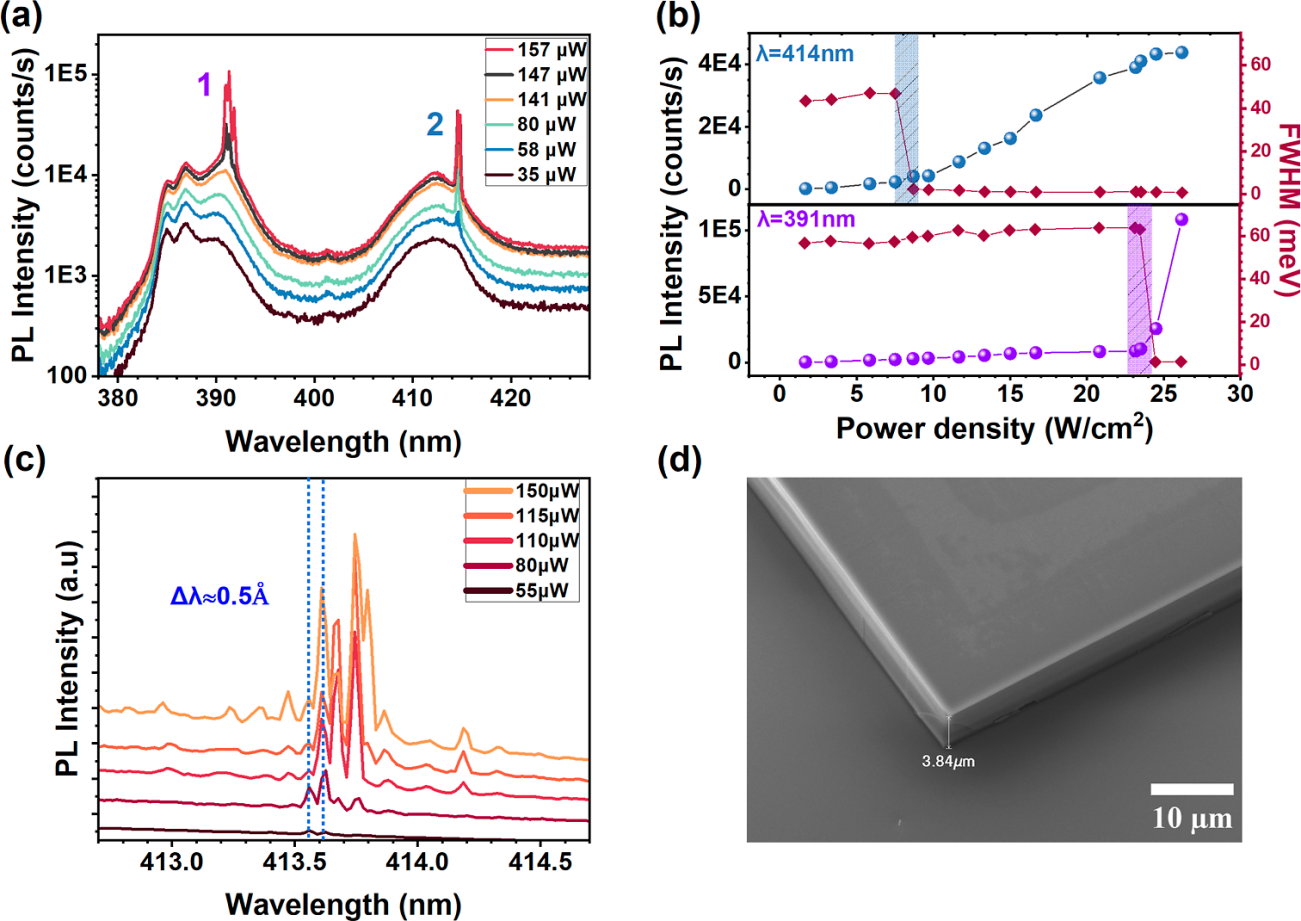
(a) PL and lasing spectra obtained at different pump powers from a MAPbCl_3_ VCSEL structure at 78K (b) PL intensity versus power density curves in spheres, for the two lasing wavelengths. The full width at half-maximum values of the emission peaks as a function of power density from below to above threshold, are also shown in rhombs. The error bars are less than 2% of indicated values on all axes. (c) Lasing spectra at different pump powers showing the spacing between longitudinal modes. (d) SEM image showing the edge of a space-confined MAPbCl_3_ single crystal sitting on a bottom DBR after having removed the top DBR. Due to the observation angle of 30°, the actual thickness is twice that measured on the image.

Another important lasing characteristic is the spacing between longitudinal modes. Figure 2c displays the longitudinal modes of the lower energy laser line, under different optical pump densities above threshold. From the typical mode linewidth of ≈0.4 Å, we infer a *Q* factor of ∼10^4^. Extracting from the graph a wavelength difference between successive modes of Δλ = 0.5 Å, we can estimate the cavity thickness *L* using the equation *L* = λ^2^/(2·Δλ·*n*_g_), where *n*_g_ is the group refractive index of the material at the lasing wavelength. Using the value of *n*_g_ = 11 from ref 18, we obtain *L* = 155 μm. This is already considerably larger than the thicknesses of 2–10 μm, typically measured with SEM on the various MAPbCl_3_ thin films. As an example, Figure 2d shows a single crystal with a thickness of ∼8 μm. Hence, the narrow mode spacing strongly suggests that the lasing oscillation does not occur in the vertical direction, but rather in the plane of the films. A possible reason for this is the very short penetration depth of a few tens of nanometers,^29^ for the 266 nm excitation used in our case, generating thus gain only at the very surface of the perovskite crystal and obstructing lasing in the vertical direction. Lateral lasing is also made possible here by the natural formation of rectangular-shaped single crystals, as the ones shown for instance in Figure 1c,d, having lateral extents that can reach several hundreds of μm. At this point, we note that dual-wavelength lasing persists even when the top DBR is removed, albeit with 2–3 times higher threshold, which strongly supports the lateral lasing picture. At the same time, the presence of the vertical cavity appears to further assist lateral lasing by lowering its threshold, most likely through enhanced waveguiding effects. Finally, it is worthwhile to mention that both laser lines are unpolarized. The mechanisms behind the lack of polarization are presently unclear and merit further investigation.

To shed light on the dual wavelength lasing of our structures, we compare in Figure 3a the μ-PL and μ-RFL spectra obtained at 78 K, from a MAPbCl_3_ single crystal after removing the top DBR. The μ-PL spectrum contains several peaks, among which we distinguish peaks 1 and 2, corresponding to the first and second lobes of Figure 2a. The intermediate PL peaks between 1 and 2, are often observed^19,26–28^ in this spectral range and are typically associated with defects. The most striking information on Figure 3a is given by the μ-RFL spectrum. Aside from the strong exciton feature next to lobe 1, marking the excitonic gap at ∼385 nm of the orthorhombic phase of MAPbCl_3_, we also note a distinct exciton feature next to the second emission lobe at ∼412 nm. This second exciton feature, which was never reported before in any MAPbCl_3_ system, can only be attributed to the coexistence within the orthorhombic phase of a “second” phase, which is present in ample quantities to give rise to such pronounced RFL signature and produce lasing. The energy position of this second feature can also explain the lower threshold of lobe 2, considering the possibility for efficient pumping via carrier transfer from the main phase of the crystal, as well as the reduced cavity losses in the transparent region of MAPbCl_3_. Incidentally, we note the presence of closely spaced Fabry–Perot oscillations in the μ-RFL spectrum, overwriting the second exciton feature and highlighting the high optical quality and parallelism of the top and bottom single crystal surfaces. Their periodicity corresponds well to a crystal thickness of 3–5 μm, in good agreement with the thicknesses measured by SEM.

**Figure 3:**
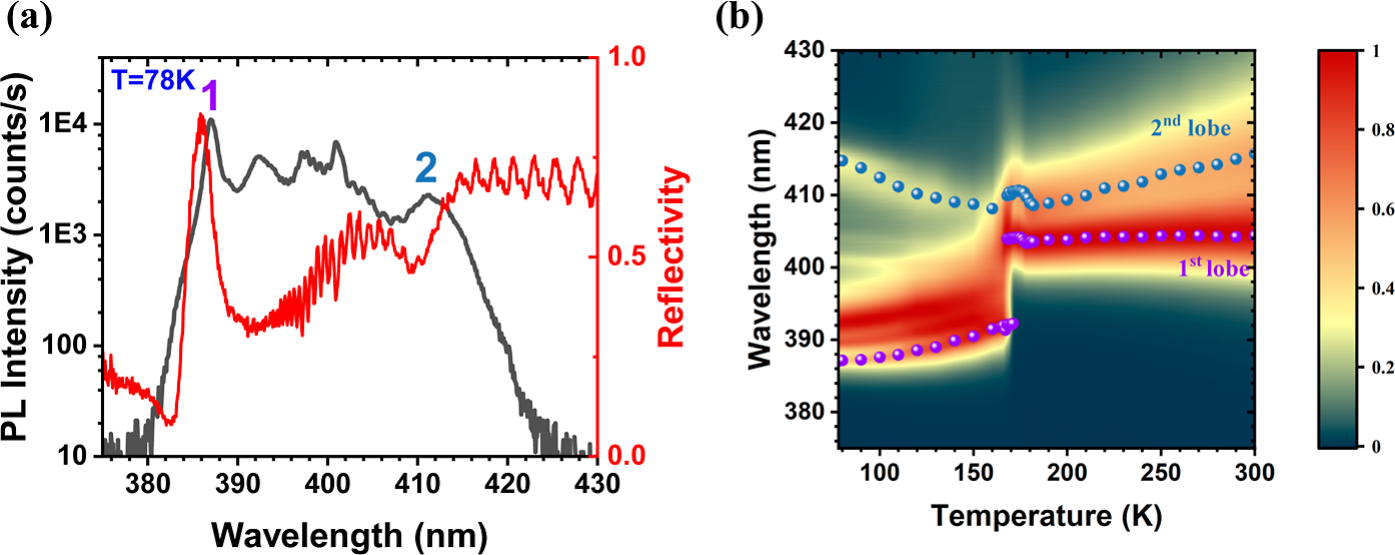
(a) Comparison of μ-PL and μ-RFL spectra on a single crystal grown on a bottom DBR. (b) Contour plot of temperature dependent μ-PL spectra on a similar crystal. The data points mark the evolution of lobes 1 and 2. The error bars on the wavelengths are less than 0.2° nm.

To gain further insight into the exciton levels of the system, we performed temperature-dependent μ-PL and μ-RFL measurements. In Figure 3b, we present the contour plot showing the evolution of the μ-PL spectra with temperature. Similar results are available for μ-RFL. The detailed μ-PL and μ-RFL spectra are presented in Figure S2 of the Supporting Information. The data points on the μ-PL contour plot mark the positions of the higher energy component of lobe 1 and of the maximum of lobe 2. With increasing temperature, lobes 1 and 2 shift variously, in line with excitonic transitions of different origin. In the orthorhombic regime (*T* < 170 K), lobe 1 redshifts continuously by ∼5 nm in the probed temperature range. At the phase transition from the orthorhombic to tetragonal, occurring at ∼170 K, lobe 1 is subject to a further abrupt redshift of ∼12 nm (∼94 meV), associated with the energy gap change in the different phases. In the “tetragonal window” between 170 and 180 K, lobe 1 undergoes an additional energy dip of ∼1–2 nm, which recovers at the “tetragonal-to-cubic” phase transition temperature of ∼180 K. In the cubic regime (*T* > 180 K), lobe 1 continues to redshift mildly, by another 1 nm, reaching an emission wavelength at room temperature of ∼405 nm. By contrast, lobe 2 behaves in a distinctly different manner. It first blueshifts in the orthorhombic regime by ∼6 nm, then also exhibits the energy dip in the “tetragonal window”, and finally redshifts vigorously in the cubic regime, by another ∼7 nm up to room temperature. The above temperature-dependence of emission lobes 1 and 2 is nicely reproduced by the exciton shifts obtained from the μ-RFL spectra, as plotted in Figure S3a of the Supporting Information.

The strong blueshift of the second lobe in the orthorhombic regime and the fact that with increasing temperature its transition energy seems to point at the cubic transition energies (c.f. Figure 3b), initially prompted us to the idea that lobe 2 could be due to emission in “cubic” inclusions, which are possibly “frozen” within the orthorhombic lattice at low temperatures. A similar hypothesis was advanced earlier, to explain the long wavelength emission at low temperatures of MAPbI_3_ thin films.^30^ However, the “cubic inclusion” scenario is unlikely in our case for two main reasons. First, a thorough analysis of both the μ-PL and μ-RFL spectra in Figure S2 of the Supporting Information, especially in the vicinity of the “orthorhombic-to-tetragonal” transition, clearly shows that lobe 2 never merges with lobe 1, but rather remains at lower energy for all temperatures. Second, if lobe 2 were merely due to “cubic” inclusions, it should normally disappear when the crystal turns cubic, which is obviously not the case as lobe 2 remains visible up to room temperature.

Lobe 2 is particularly prominent in the thin MAPbCl_3_ single crystals used in this work, strongly suggesting a correlation with the space-confined growth method and possibly with the crystal size. To illustrate this, we compare in Figure 4, the μ-PL and μ-RFL spectra at different temperatures, of a thin MAPbCl_3_ single crystal grown on a bottom DBR as described in Experimental Methods, and a free-standing bulk MAPbCl_3_ single crystal grown by the inverse temperature crystallization method, detailed in Materials and Methods of Supporting Information.^6,17,31^ The full temperature-dependent spectra and related exciton shifts of the bulk single crystal, are presented in Figures S4 and S3b of the Supporting Information, respectively. The main difference between the growth methods used for the thin and bulk single crystals, is that in the former case, a finite quantity of the solution is spread on the DBR and diminishes continuously as the solvent evaporates in the oven, whereas in the latter case, the solution is always present during the crystal growth. Focusing first on the μ-RFL spectra of Figure 4, we observe a very pronounced lobe 2 feature in the thin crystal at all temperatures, while lobe 2 appears completely absent in the bulk reflectivity. A similar but less firm conclusion can be reached by comparing the μ-PL spectra of Figure 4. In the orthorhombic phase (*T* = 78 K), the emission of lobe 2 appears significantly weaker in the bulk single crystal compared to the thin case, by at least 1 order of magnitude. Similarly, at higher temperatures, lobe 2 is visible in the thin crystal in the form of a distinct peak, while in the bulk it is clearly much weaker, possibly “hiding” in the asymmetric emission profile. In other words, even though the second phase associated with lobe 2 is particularly pronounced in the thin space-confined single crystals of this work, it is likely that this second phase is more generally present in the MAPbCl_3_ system, but to a variable degree depending on the specifics of the growth conditions. In line with this, we note that lobe 2 is also detectable in thin single crystals grown either on “uncovered” bottom DBR regions (without a top DBR) or in-between various other templates, such as Silicon or Sapphire (c.f. Figure S5 of the Supporting Information). In addition, lobe 2 is also traceable in the PL spectra of prior works on MAPbCl_3_ single crystals, where it was attributed either to bound excitons at defect levels^6,17,31^ or to reabsorption phenomena.^6,17,31^

**Figure 4:**
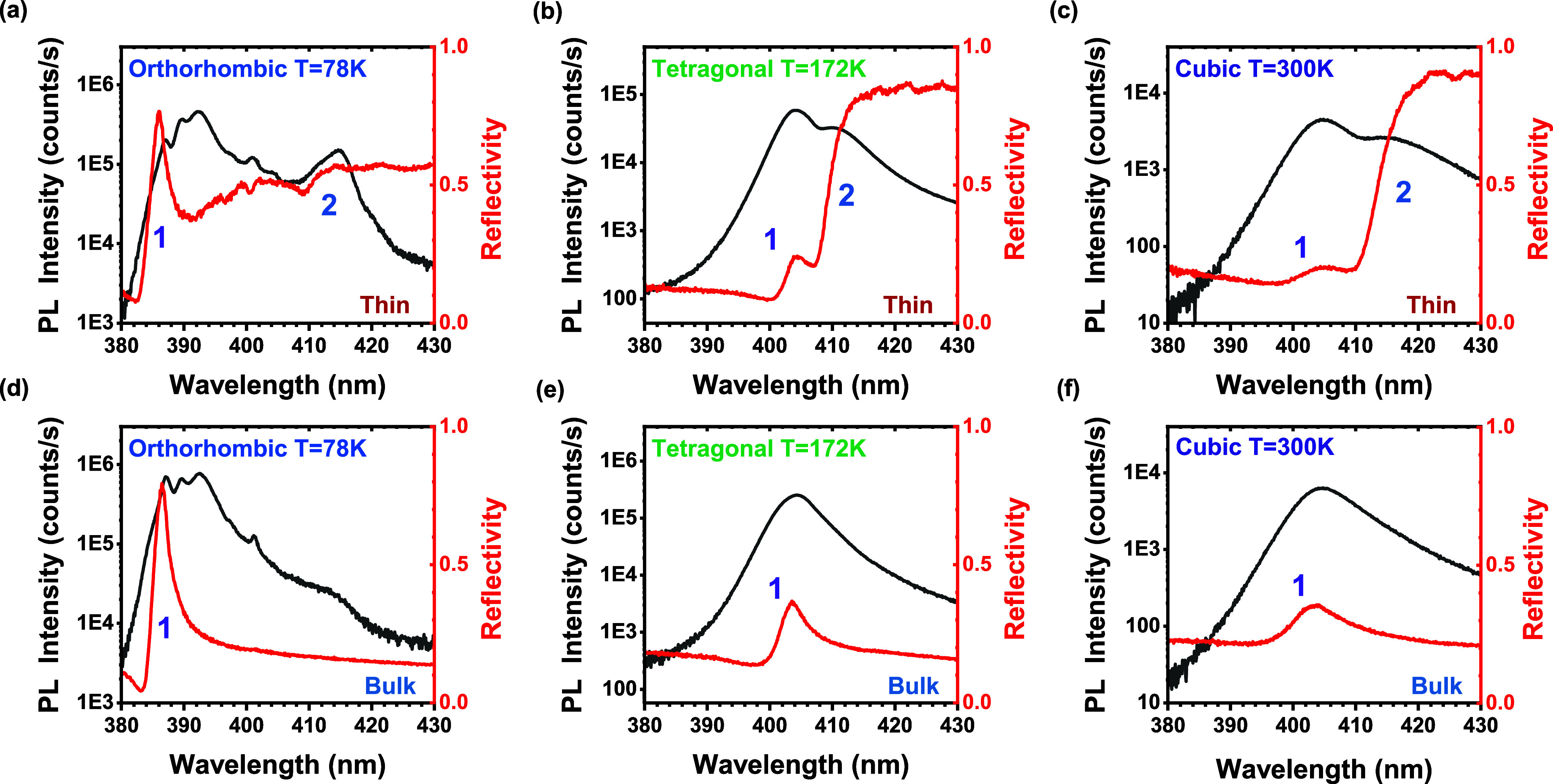
Comparison of μ-PL and μ-RFL spectra at three different temperatures corresponding to distinct crystal phases, of a thin MAPbCl3 single crystal grown on a DBR (a–c), and a bulk MAPbCl3 single crystal (d–f).

In view of these exciting results, we have sought further evidence about the second phase, based on structural characterization methods. In Figure 5a, we present a 5 μm × 5 μm AFM image, taken from the surface of a ΜΑPbCl_3_ thin crystal grown on a Si(100) substrate, using the space-confined method of this work. Qualitatively similar results have been obtained on larger scans such as 10 μm × 10 μm, but also on other templates as well, crystalline or amorphous, that will be presented elsewhere. Somewhat unexpectedly, considering the extreme simplicity of the growth method, we observe the formation of an ordered and ultrasmooth MAPbCl_3_ surface, returning a remarkable root-mean-square (rms) roughness value of 0.2 nm over the whole 5 μm × 5 μm area. Such low rms-value can be encountered only in high-quality atomically flat epitaxial samples, and what makes it even more impressive, is that a good fraction of it is due to the step-like periodic structure, which is visible over the whole surface. As can be deduced from the AFM profile of Figure 5c, the periodic structure has a period of ∼250 nm in the direction perpendicular to the steps, and a step height of ∼0.6 nm. The latter coincides practically with the c-lattice parameter of MAPbCl_3_ of 0.5675 nm, strongly suggesting that the periodic structure is due to the spontaneous formation of atomic steps on the surface, induced by some misorientation of the perovskite lattice. It should be noted that the steps are oriented either perpendicular or parallel to the rectangular edges of the thin crystallites, seen in Figure 1c,d. Perhaps the most interesting observation that can be made on Figure 5a, with direct ramifications to this work, is that the MAPbCl_3_ surface appears to be “decorated” with quantum-dot-like nanostructures, as emphatically depicted in the zoomed-in AFM area of Figure 5b. These quantum dots (QDs) tend to nucleate at the edges of atomic steps and their typical dimensions can be extracted by AFM profiles, as the one shown in Figure 5d. The outcome of this analysis, over the whole 5 μm × 5 μm scan area, is presented in the histograms of Figure 5e,f, suggesting that the QD heights range between 0.6 and 1.6 nm with a mean value of 0.9 nm, while the QD diameters between 15 and 45 nm with a mean value of 32 nm. The corresponding QD density in this sample is ∼8.5 particles/μm^2^. By examining closely several AFM images, including that of Figure 5b, these QD-like nanostructures can be also discerned beneath the surface of MAPbCl_3_, suggesting that they are not only a surface effect, but exist also in the bulk of the crystals. This realization allows us to advance the hypothesis that these QD-like nanostructures correspond to the “second phase”, observed so vividly in the optical experiments. An additional observation, corroborating with the QD-hypothesis, is presented in Figure S6 of the Supporting Information, where the sample inhomogeneity in terms of QD structural parameters can be correlated with significant variations in the second exciton feature from one spot to another.

**Figure 5:**
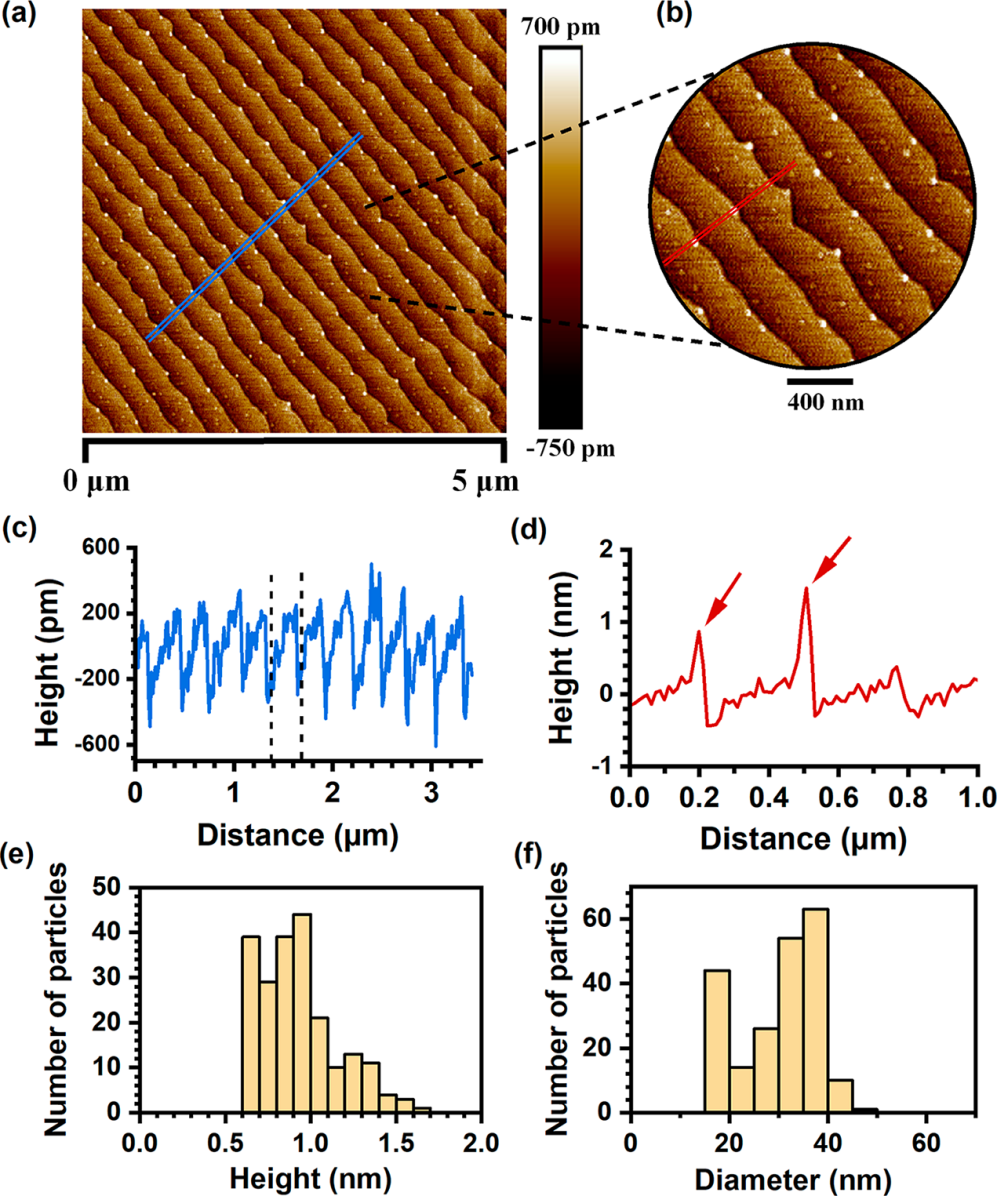
(a) AFM image showing a very smooth and ordered MAPbCl_3_ surface with a periodic atomic-step-like structure. (b) Zoomed-in AFM image extracted from the image of panel (a), showcasing QD-like nanostructures nucleating at the edges of atomic steps. By careful inspection, one can also discern the existence of QDs beneath the crystal surface. (c) Height profile along the blue line of panel (a), allowing measurements of the period and step height of the periodic structure. (d) Height profile along the red line of panel (b), where the spikes indicated by arrows correspond to QDs that fell exactly at or near the profile line. (e,f) Histograms of QD heights and diameters produced by AFM analysis over the 5 μm × 5 μm scan area of the sample. The corresponding QD density is ∼8.5 particles/μm^2^.

To possibly extract information about the crystalline nature of the second phase, we performed precession X-ray diffraction (XRD) experiments as a function of temperature on selected single-crystals of MAPbCl_3_ taken from the two growth batches (thin film and bulk). Experimental details about Single-Crystal XRD can be found in the Supporting Information. The most visual difference between the patterns of the two crystals shown in Figure S7 of the Supporting Information is the diffraction quality, with the thin crystal obtained from the film showing a much “cleaner” pattern compared to the bulk crystal, partially due to the diffraction from a second crystal in the latter case. On the other hand, the bulk crystal exhibits an overall stronger diffraction intensity, possibly due to the larger volume of the crystal. These visual characteristics, however, do not reflect any significant differences in the temperature evolution of the unit cell (cf. Table S1 of the Supporting Information).^32–34^ The feature, however, that seems to be relevant in the patterns of the two crystals, is the relative intensity of the supercell reflections with respect to the intensity of the basic perovskite subcell (defined by the 1 × 1 × 1 cubic perovskite cell and its reflections, as seen in Figure S7). The stronger presence of the subcell reflections in the thin film, suggests that the crystal retains more of its subcell structure, or in other words, the crystal tends to be “more cubic” hinting toward the possibility that there is some frozen “cubic-like” subcell underneath the observed supercell, a hypothesis often used by the perovskite spectroscopy community.^30^ The presence of such a secondary phase could well justify the appearance of the second exciton resonance (lobe 2) due to the different electronic structure, but this is a statement that is not possible to be unequivocally resolved by X-ray diffraction.^35–38^ Another feature that is also associated with the subcell/supercell diffraction intensity, concerns very specific diffraction planes at the lowest diffraction angles. These reflections are crystallographically defined only at 100 K, while at higher temperatures “melt” into polycrystalline rings. We attribute this effect to dynamic disorder, which is a well-known phenomenon at the temperature range under consideration.^39–41^ Dynamic disorder cannot account for the optical results in any obvious way, since both types of crystals exhibit a very similar “melting” effect. However, together the two unusual phenomena discussed above, provide further indication that secondary perovskite-associated phases are likely present beneath the apparent periodic lattice, thus supporting the observed second exciton resonance in MAPbCl_3_. A possible explanation why the XRD experiments provide only indirect evidence about the second phase is probably related to the small volume ratio of the second-phase inclusions in the crystal. An order of magnitude estimate of the volume ratio can be obtained as follows: assuming that each atomic terrace of the periodic structure contains only QDs at the edges of the atomic steps and that this surface motif is repeated throughout the volume of the crystal, then it can be shown that the volume ratio is essentially limited by the surface coverage of the QDs, which based on parameters mentioned in the discussion of Figure 5, does not exceed the 1–2%, explaining how the XRD method is relatively insensitive to the second phase.

## 4 Conclusions

The observation of dual-wavelength lasing at 78 K in a vertical MAPbCl_3_ cavity, led us to the discovery of a second, previously unreported, crystal phase within the orthorhombic lattice of MAPbCl_3_. This second phase manifests itself by a marked excitonic transition in microreflectivity, whose optical signature can be traced all the way up to room temperature, making unlikely that this second phase consists simply of cubic inclusions. Even though our experiments clearly suggest a correlation of the second phase with the growth method and possibly with the size of the single crystal, it is likely that this second phase is generally present in the MAPbCl_3_ system, but to a varying degree. The second phase is likely associated with the quantum dot-like nanostructures, revealed on the surface of the MAPbCl_3_ crystals by AFM measurements. Further studies are necessary to shed light on the precise composition and crystal structure of these second phase inclusions in MAPbCl_3_ single crystals, including high-resolution electron transmission microscopy, micro-PL with submicron spatial resolution, and pair distribution function measurements by X-ray diffraction. The conclusions drawn from this study bring about new understanding of the halide perovskites family and initiate a path toward the next generation of photonic perovskite devices in the near UV spectral region.

## Supplementary Material



## Notes

Additional lasing spectra, detailed temperature-dependent PL and RFL measurements, influence of template and sample inhomogeneity with the second exciton feature, X-ray diffraction data, description of materials and methods (PDF)
