# Supplementary material for: Dual-Wavelength Lasing Due to Second Phase Inclusions in MAPbCl3
Source: J Phys Chem C Nanomater Interfaces. 2025 Nov 12;129(47):21016–23. doi: 10.1021/acs.jpcc.5c05466 (PMC12670514; doi:10.1021/acs.jpcc.5c05466)
Supplement: Supplementary file 1 [file jp5c05466_si_001.pdf]

Supporting Information

Dual-wavelength lasing due to second phase inclusions in $MAPbCl_3$

Christina Siaitanidou[1,2], Violeta Spanou[3], Nikolaos G. Chatzarakis[1,2], Panagiotis Oikonomopoulos[4], Giannis S. Papaefstathiou[4], Katerina Tsagaraki[2], Eric Delamadeleine[5], Constantinos C. Stoumpos[1,6], Nikolaos T. Pelekanos[1,2]**

[1]Department of Materials Science & Engineering, University of Crete, P.O. Box 2208, 70013 Heraklion, Greece

[2]Microelectronics Research Group, IESL-FORTH, 70013 Heraklion, Greece

[3]Department of Chemistry, University of Crete, P.O. Box 2208, 70013 Heraklion, Greece

[4] Laboratory of Inorganic Chemistry, Department of Chemistry, National and Kapodistrian University of Athens, 15771 Athens, Greece

[5]Univ. Grenoble-Alpes, CEA, Grenoble INP, IRIG, PHELIQS, 38000 Grenoble, France

[6]Photonics of Crystals Laboratory, Saint Petersburg State University, Ulyanovskaya d.1, St. Petersburg 198504, Russia

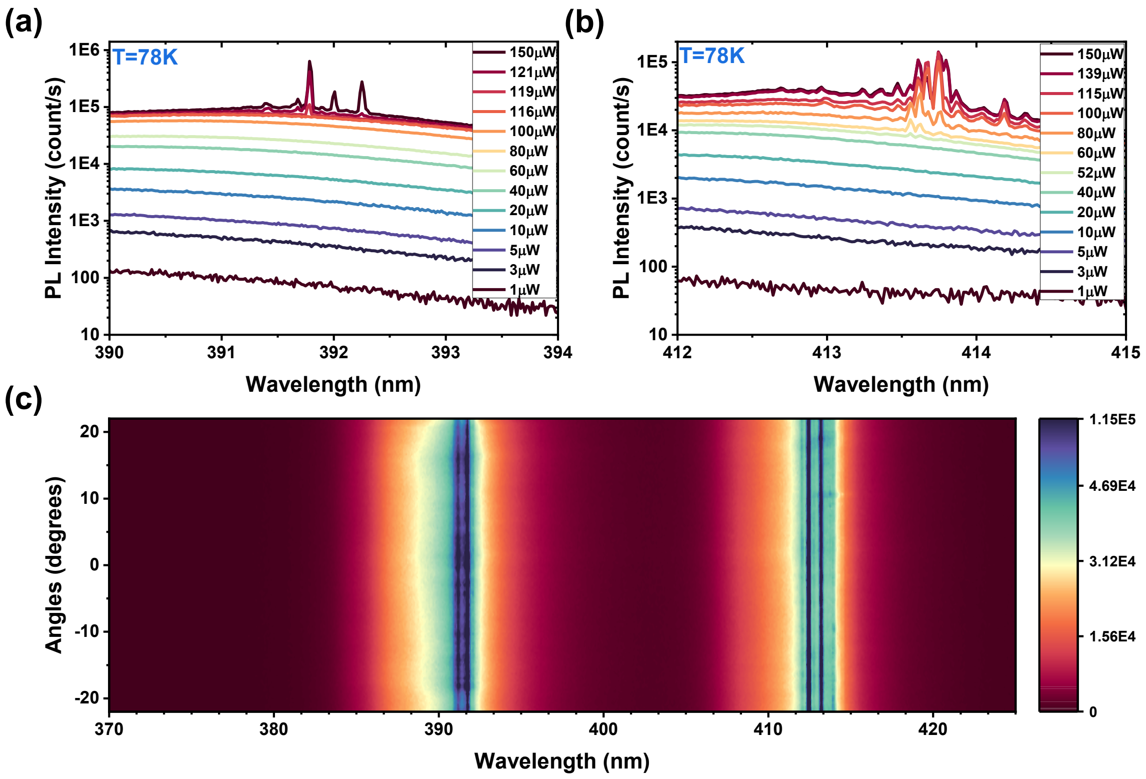

Figure S1. (a)-(b) Emission spectra with increasing pump power from *below* to *above* threshold, for the two laser lines corresponding to lobes 1 and 2, obtained on a different $MAPbCl_3$ VCSEL structure using a 2400 gr/mm grating. (c) Angle-resolved k-space image obtained from the same laser structure, while in dual-wavelength lasing conditions, using a 600 gr/mm grating.

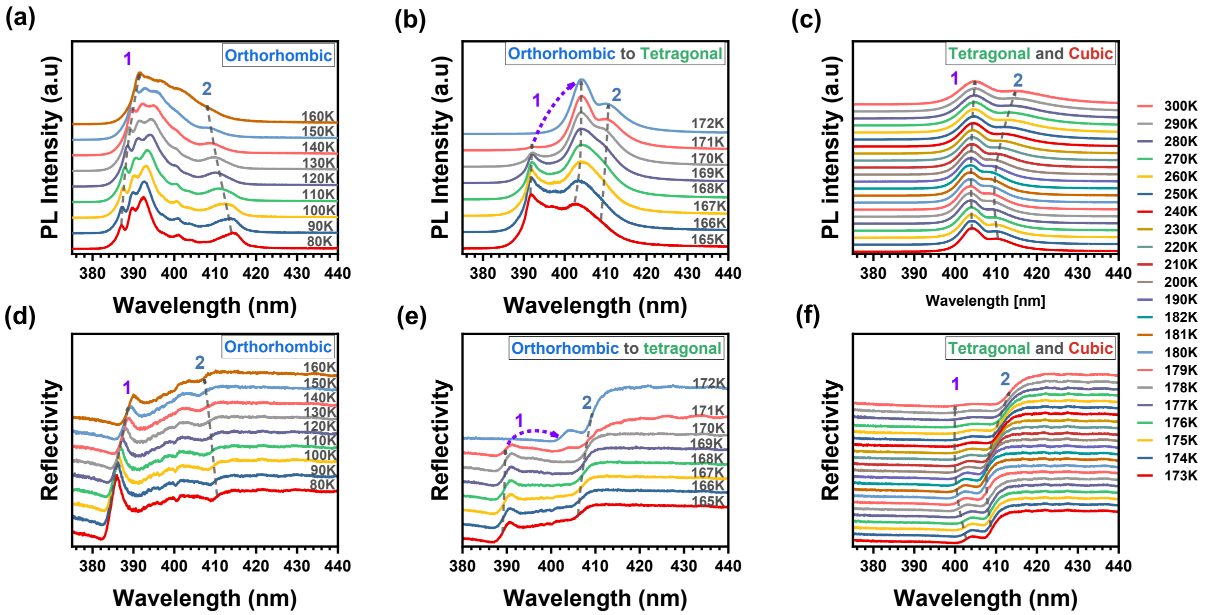

Figure S2. Comparison of temperature dependent μ-PL (a)-(c) and μ-RFL (d)-(f) spectra obtained in different temperature ranges on a thin $MAPbCl_3$ single crystal grown on a bottom DBR.

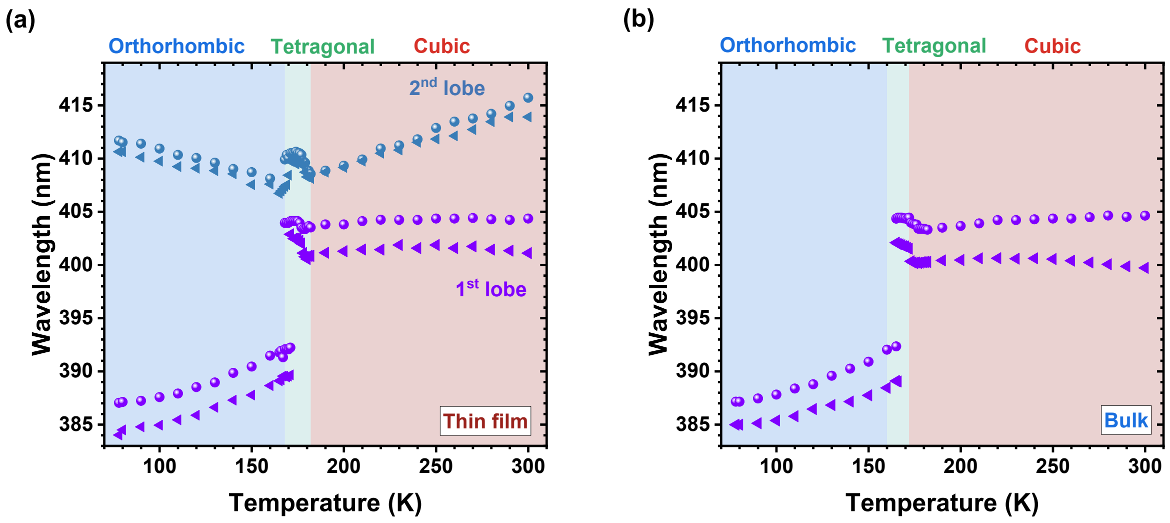

Figure S3. Transition energies extracted from temperature dependent μ-PL and μ-RFL spectra of (a) a thin $MAPbCl_3$ single crystal grown on a bottom DBR, and (b) a bulk $MAPbCl_3$ single crystal. The spheres correspond to μ-PL data points, while the triangles to μ-RFL. Please note the absence of the 2nd lobe in the bulk single crystal case.

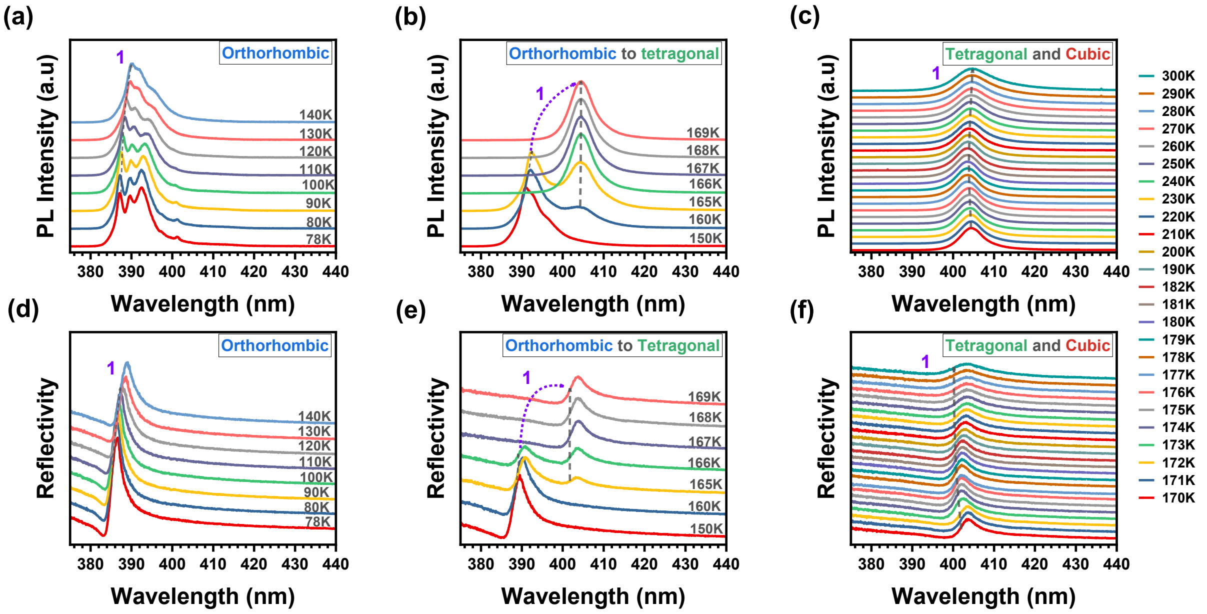

Figure S4. Comparison of temperature dependent μ-PL (a)-(c) and μ-RFL (d)-(f) spectra obtained in three different temperature ranges on a bulk $MAPbCl_3$ single crystal.

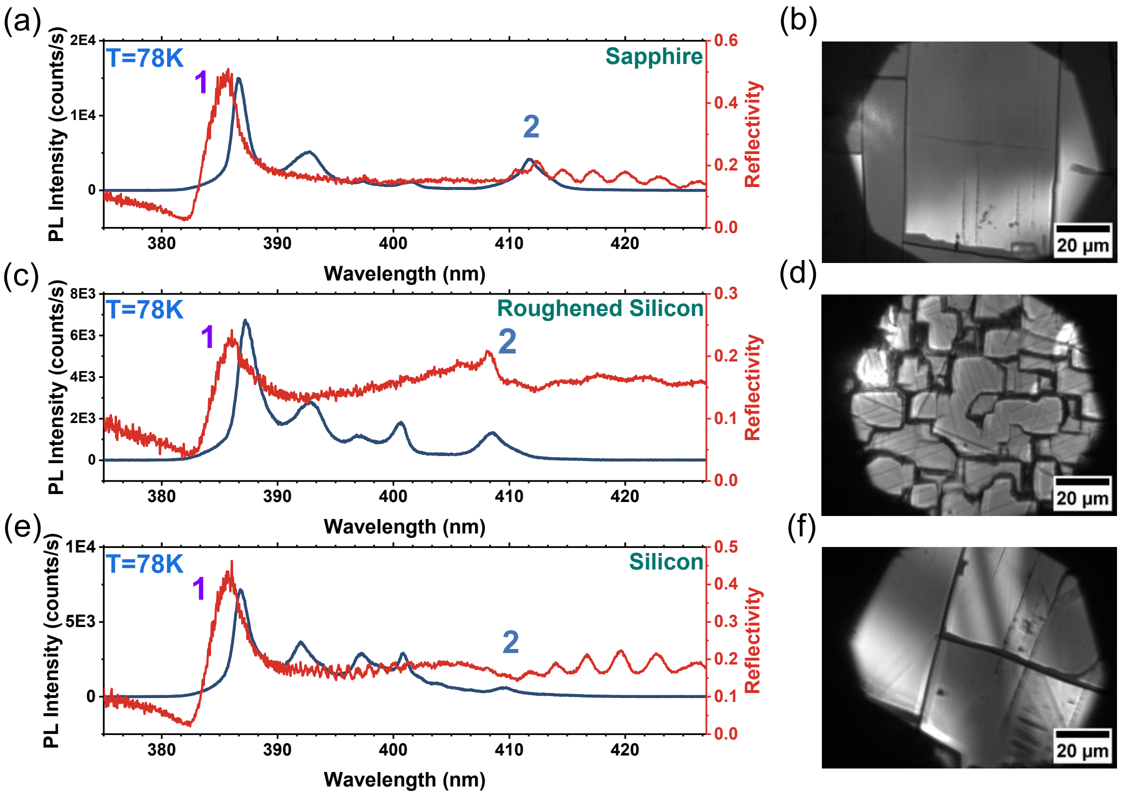

Figure S5. Comparison of optical spectra at T=78K and optical microscopy images of $MAPbCl_3$ thin crystals, deposited on various templates such as (a,b) polished c-axis Sapphire, (c,d) unpolished (100) Silicon wafer, (e,f) epi-ready (100) Silicon wafer. Both exciton features are visible in the spectra, regardless of the template used.

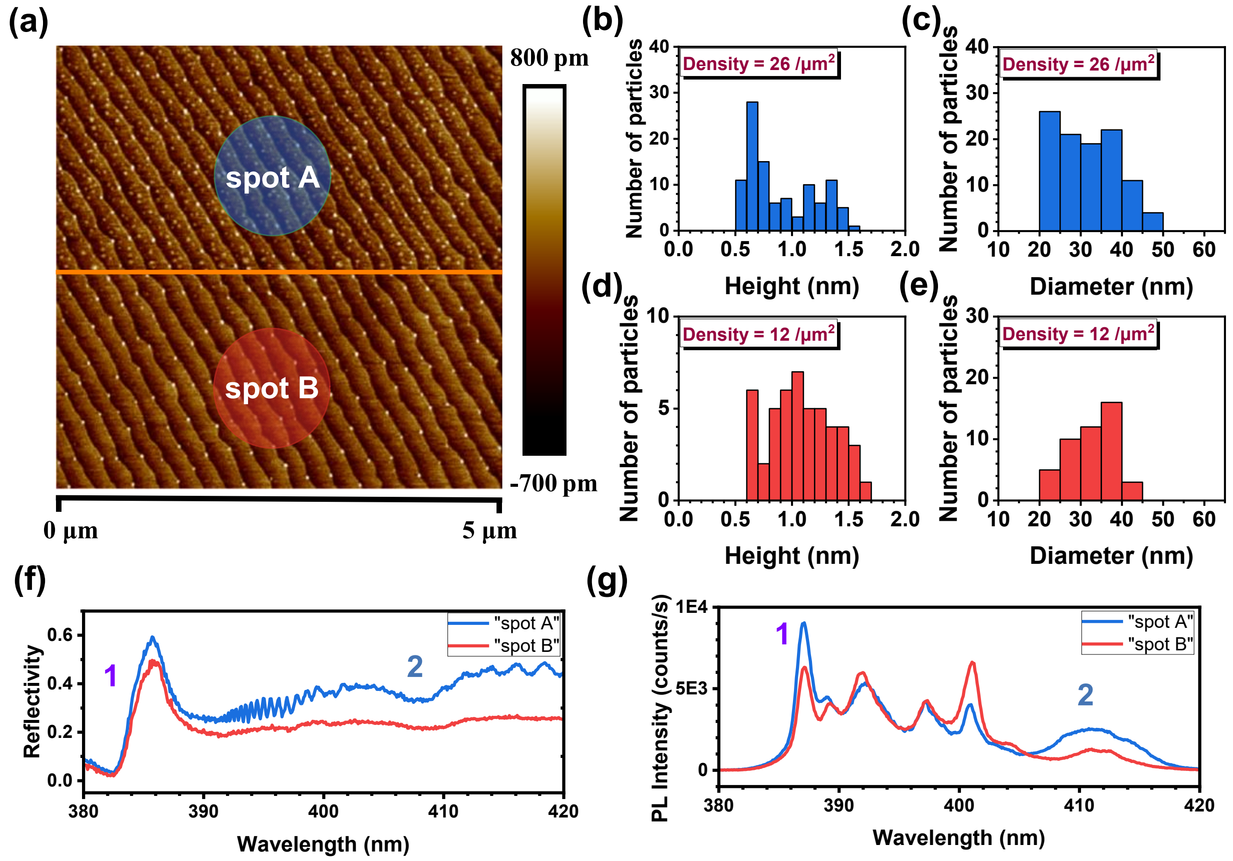

Figure S6. (a) AFM images highlighting two regions of a $MAPbCl_3$ crystal (spot A and spot B) with varying QD densities. The corresponding height and diameter histograms over 2 µm x 2 µm areas, are shown in (b) and (c) for spot A, and in (d) and (e) for spot B, respectively. Panels (f) and (g) compare the reflectivity and PL spectra from different positions of the same sample, suggesting a clear correlation of the intensity and spectral position of the second exciton feature, with sample inhomogeneity in terms of QD structural characteristics.

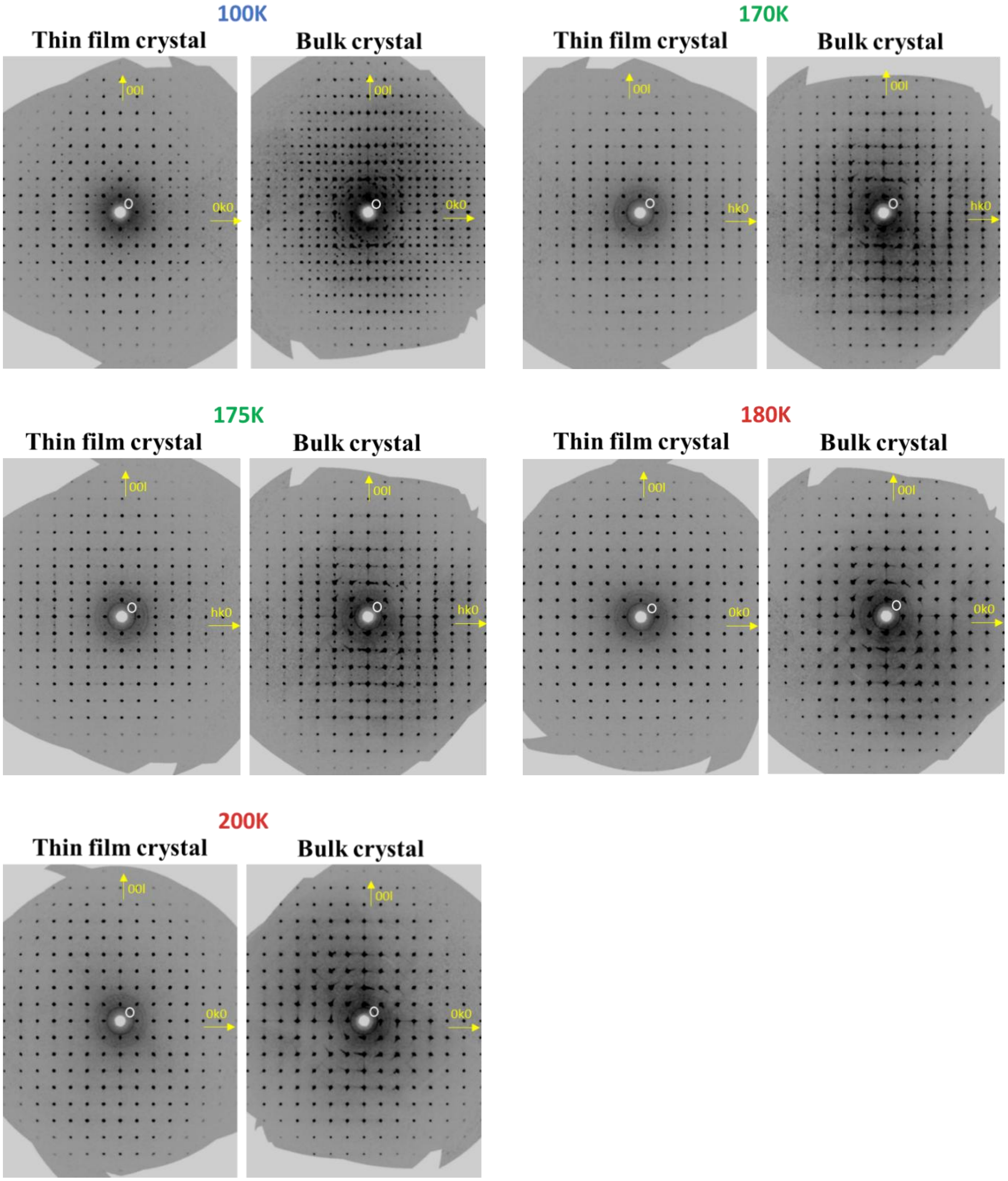
100K
Thin film crystal
Bulk crystal
00l
0k0
170K
Thin film crystal
Bulk crystal
00l
hk0
175K
Thin film crystal
Bulk crystal
00l
hk0
180K
Thin film crystal
Bulk crystal
00l
0k0
200K
Thin film crystal
Bulk crystal
00l
0k0

Figure S7. Reconstructed precession images at different temperatures, generated from the experimental X-ray diffraction data for the two $MAPbCl_3$ crystals at the indicated lattice directions. The thin crystal exhibits a much cleaner pattern, partly because of the presence of a secondary crystal in the pattern of bulk $MAPbCl_3$, which at 200K indexes at 6.9% of the total reflections. The white circle, which at 100K represents the (011) plane, monitors the loss with increasing temperature of long-range ordering (crystal spots vs polycrystalline rings) as a consequence of the dynamic disorder in the crystals.[1–3]

Table S1. Crystallographic parameters for the $MAPbCl_3$ single-crystals at the indicated temperatures.

Lattice parameter/Temperature	100K	170K	175K	180K	200K
Thin film crystal					
Lattice	Primitive orthorhombic	Primitive orthorhombic	Primitive orthorhombic	Primitive cubic	Primitive cubic
Indexing (%)	95.70	96.73	97.22	99.54	99.26
a (Å)	11.2127(3)	7.9969(3)	8.0010(3)	5.66339(9)	5.66789(10)
b (Å)	11.3073(3)	7.9982(3)	7.9994(3)	5.66339(9)	5.66789(10)
c (Å)	11.3491(4)	11.3314(5)	11.3325(5)	5.66339(9)	5.66789(10)

V (Å^3)	1438.89(8)	724.76(5)	725.32(5)	181.648(5)	182.081(5)
V_{norm} (Å^3)	(/8) 179.22	(/4) 181.71	(/4) 181.54	(/1)181.58	(/1) 182.08
Bulk crystal					
Lattice	Primitive orthorhombic	Primitive orthorhombic	Primitive orthorhombic	Primitive cubic	Primitive cubic
Indexing (%)	76.81	78.32	82.86	90.42	91.22
a (Å)	11.2116(4)	7.9816(3)	7.9815(3)	5.66270(9)	5.66754(10)
b (Å)	11.2709(4)	8.0178(3)	8.0143(3)	5.66270(9)	5.66754(10)
c (Å)	11.3467(4)	11.3581(5)	11.3520(4)	5.66270(9)	5.66754(10)
V (Å^3)	1433.82(9)	726.85(5)	726.15(4)	181.581(5)	182.047(5)
V_{norm} (Å^3)	(/8) 179.86	(/4) 181.19	(/4) 181.33	(/1)181.65	(/1) 182.05

Discussion on the crystal structure of $MAPbCl_3$

Although there are many studies on the temperature evolution of the crystal structure of $MAPbCl_3$ following its original discovery by Weber,[4] there are still today ambiguities about the crystal structure of the compound. Among several published crystal structures, contradictory results regarding the low temperature structure of $MAPbCl_3$ have appeared, varying between the original assignment of a 2 x 2 x 2 supercell[4,5] and a recent reevaluation suggesting a √2 x √2 x 2 orthorhombic cell, analogous to the γ-phase of $MAPbBr_3$ and $MAPbI_3$. Such a variety in the structural determination implies some special complexity in the system, which may be associated with the lattice dynamics, as $MAPbCl_3$ has a more ionic character compared to the heavier halides,

or it could simply derive from the accidental parity on the electron count on both ions occupying the A and X sites of the perovskite structure, thus precluding the accurate assignment of the space group.

The single crystal patterns obtained from our datasets on both types of thin and bulk single crystals seem to support the suggestion of Alvarez-Galván *et al.,*[6] that there is indeed a further complexity in the evolution of the structure at low temperature. We also support their assignment since it is the only one that falls within the theoretical phase transition models[7–10] of perovskites. Even though a complete structure determination is beyond the scope of the present work, the comparison of the diffraction patterns obtained from the two types of crystals at set temperatures (Figure S7 and Table S1 of the Supporting Information), reveals some important characteristics that may be of relevance to the evolution of lasing and the presence of a second strong excitonic feature that evolves in the post phase-transition temperature range.

Based on the reflection indexing of our data, we find that at 100K, both patterns can be indexed with a 2 x 2 x 2 supercell, whereas the sets collected at 170K and 175K can be indexed with a √2 x √2 x 2 supercell. At 180K and 200K, both sets of patterns acquire the ideal cubic structure. Although small variations in the lattice parameters can be observed (see Table S1), their significance does not seem relevant to the observed optical properties.

Looking at the intensities of some specific reflections, the difference in intensity becomes obvious in the patterns of the two crystals. Considering the 041 and 022 reflections at 100K as an example, we find that their relative intensity changes significantly, with the bulk crystal having a relative ratio of ~ 1:20 and the thin crystal having a ratio of ~1:10, i.e. an intensity difference corresponding to a factor of 2. This ratio approximately applies to many of the subcell/supercell pairs in the 0kl reconstructed layers (see Figure S7) and also persists in the intermediate unit cell

between 170-175K. This difference in intensity cannot be easily attributed to the structural features associated with a single unit cell and it is intriguing to consider that some of the missing remnant intensity could be attributed to a hidden subcell, masked underneath the indexed supercell.

Moreover, the low angle peaks exhibit unexpected behavior at different temperatures. Specifically, the reflections that are associated with the highest resolution spots (011 at 100K, indicated as white circles in Figure S7), form well-defined spots at 100K in both crystals, which transform to polycrystalline diffraction rings at higher temperatures, persisting across the whole examined temperature range (170-200K). To the best of our knowledge, this loss of long-range ordering has not been observed previously in single-crystal diffraction data, but given the specific temperature window, we can rather confidently assume that it is associated with the well-known effect of dynamic disorder in the perovskite lattice.[1–3]

Materials and Methods

Solution preparation

For the synthesis of perovskite single crystals, a 0.7 M MAPbCl3 solution is prepared by dissolving 7 mmol of PbCl2 (1.946 g) and 7 mmol of MACl (0.473 g) in a solvent mixture of 5 mL of DMSO and 5 mL of DMF. The resulting mixture is stirred at room temperature, until it becomes translucent, indicating a complete dissolution of the precursors. As a next step, the solution is filtered using a PVDF 0.45 μm filter to get rid of any solid impurities present.

Synthesis of thin Single crystals using the space confined method

For the crystallization of space-confined MAPbCl3 single crystals, a small amount of a few μl of the solution is dropped and spread on a bottom DBR. Then a top DBR is placed face-down on top, and by applying some pressure the solution is uniformly spread and fills the air gap between

the DBRs. By placing the top DBR, the crystallization of single crystals is space confined. Then the sample is put in a preheated oven at 60°C where the crystallization takes place. The sample remains in the oven for 24hrs to ensure that all solvents are evaporated.

Synthesis of bulk Single crystals

Bulk MAPbCl3 single crystals are prepared according to the inverse temperature crystallization (ITC) method.[11–13] The same solution of 0.7 M MAPbCl3 is used for this synthesis. The solution is gradually heated up to 70°C in an oil bath. After 24 h, the transparent, mm-sized cubic crystals are removed from the solution and are press-dried using paper towels.

For the crystallization of the two systems, the same solution is used. However, there is a major difference between the two methods. In the space-confined method, the solvents are continuously evaporated inducing crystallization, whereas in the bulk case the solution is always present, and the temperature is the main driving mechanism of the crystallization. This explains the different morphology of the obtained crystals, which are thin rectangles in the space-confined method and thick cube-like crystals in the ITC method, as visible in Figure S8.

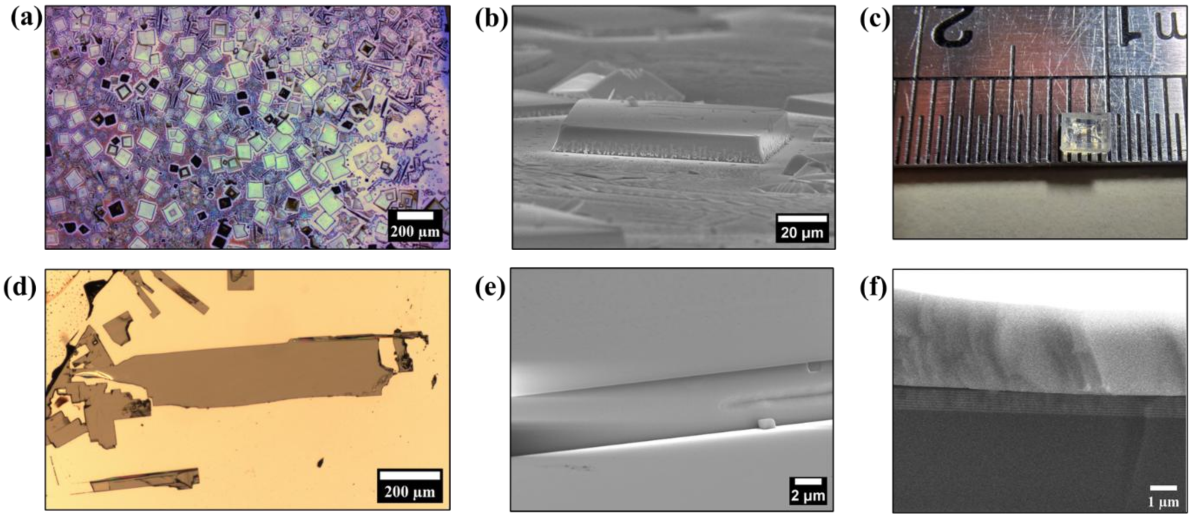

Figure S8. (a) Optical microscope image showing $MAPbCl_3$ thin single crystals grown by dropping a few µl of solution on a bottom DBR/sapphire substrate and then introduce it uncapped in the oven at 60ºC for 10 mins. (b) A large tilting-angle SEM image showing a thin single crystal grown uncapped, with the crystallization method mentioned in (a). (c) Optical image of mm-sized $MAPbCl_3$ bulk single crystal grown by the inverse temperature crystallization method. (d) Optical microscope image showing a rectangular shaped thin single crystal grown with the space-confined method. (e) A large tilting-angle SEM image showing a single crystal grown with the space-confined method of this work, in good adhesion with the underneath template. (f) A cross-section SEM image showing the thickness profile of a thin single crystal grown on a DBR, after removing the other DBR.

Single-Crystal X-Ray diffraction

Single-crystals produced by the two methods were measured at 100K, 170K, 175K, 180K and 200K in order to monitor the structural changes above, below and near the phase transition of MAPbCl3. A single-crystal from the antisolvent method (0.065 x 0.100 x 0.112 mm3) and a single crystal from the encapsulation method (0.025 x 0.085 x 0.086 mm3) were mounted on a Bruker

D8 Venture diffractometer equipped with an IμS 3.0 Mo Kα (λ = 0.71073 Å) source operating at 50kV and 1.4 mA, Photon-III CMOS area detector and a four-circle kappa goniometer. The temperature was controlled using an Oxford Cryostream 800 cryostat. In both cases, the data collection strategy was estimated for the indexing at 100K and the strategy profile was kept constant across the whole temperature range.

Data analysis was performed using Crysalis Pro (version 40.71a). The unit cell parameters were determined based on unit cell indexing for the complete data set and the precession images of selected diffraction layer were performed using the "unwarp" function.

Atomic Force Microscopy

The AFM images were obtained using in tapping mode Bruker's Dimension Icon model, having noise levels in the sub-angstrom range for the Z-axis, and angstroms in XY.

Optical Characterization

The μ-PL and μ-RFL spectra were obtained using an Acton Spectra Pro 500i spectrograph with a 600 g/mm grating blazed at 300 nm, equipped with a liquid nitrogen-cooled charge-coupled device camera (CCD). In most μ-PL experiments, we used a pulsed 266 nm laser (Nanolase NU-10210-101) with 7.58 kHz rep rate and 0.51 ns pulse width, as excitation source. The beam was focused through a quartz aspheric lens leading to a 60x10 μm^2 spot on the sample surface. The corresponding μ-PL emission was collected by the same lens and was focused on the spectrograph slits by a 10 cm lens. In the particular μ-PL spectrum of Fig.3(a), a frequency-doubled 365 nm femtosecond laser was used instead, with 80 MHz rep rate and 120 fs pulse width. The μ-RFL spectra were obtained with a 150 W Xe lamp (Hamamatsu E7536) and the light was focused on

the surface of the crystals with a x10 objective lens (Olympus UPlanFl 10x/0.30). The μ-RFL spectra were normalized using the reflectivity spectrum of an aluminum mirror sitting next to samples.

References

(1) Guo, Y.; Yaffe, O.; Hull, T. D.; Owen, J. S.; Reichman, D. R.; Brus, L. E. Dynamic Emission Stokes Shift and Liquid-like Dielectric Solvation of Band Edge Carriers in Lead-Halide Perovskites. *Nature Communications 2019 10:1* **2019**, *10* (1), 1–8. https://doi.org/10.1038/s41467-019-09057-5.

(2) Yaffe, O.; Guo, Y.; Tan, L. Z.; Egger, D. A.; Hull, T.; Stoumpos, C. C.; Zheng, F.; Heinz, T. F.; Kronik, L.; Kanatzidis, M. G.; Owen, J. S.; Rappe, A. M.; Pimenta, M. A.; Brus, L. E. Local Polar Fluctuations in Lead Halide Perovskite Crystals. *Phys Rev Lett* **2017**, *118* (13), 136001. https://doi.org/10.1103/PHYSREVLETT.118.136001/FIGURES/3/MEDIUM.

(3) Guo, Y.; Yaffe, O.; Paley, D. W.; Beecher, A. N.; Hull, T. D.; Szpak, G.; Owen, J. S.; Brus, L. E.; Pimenta, M. A. Interplay between Organic Cations and Inorganic Framework and Incommensurability in Hybrid Lead-Halide Perovskite CH3NH3PbBr3. *Phys Rev Mater* **2017**, *1* (4), 042401. https://doi.org/10.1103/PHYSREVMATERIALS.1.042401/FIGURES/4/MEDIUM.

(4) Poglitsch, A.; Weber, D. Dynamic Disorder in Methylammoniumtrihalogenoplumbates (II) Observed by Millimeter-wave Spectroscopy. *J Chem Phys* **1987**, *87* (11), 6373–6378. https://doi.org/10.1063/1.453467.

(5) Chi, L.; Swainson, I.; Cranswick, L.; Her, J. H.; Stephens, P.; Knop, O. The Ordered Phase of Methylammonium Lead Chloride CH3ND3PbCl3. *J Solid State Chem* **2005**, *178* (5), 1376–1385. https://doi.org/10.1016/J.JSSC.2004.12.037.

(6) Alvarez-Galván, M. C.; Alonso, J. A.; López, C. A.; López-Linares, E.; Contreras, C.; Lázaro, M. J.; Fauth, F.; Martínez-Huerta, M. V. Crystal Growth, Structural Phase Transitions, and Optical Gap Evolution of CH 3 NH 3 Pb(Br 1-x Cl x) 3 Perovskites. *Cryst Growth Des* **2019**, *19* (2), 918–924. https://doi.org/10.1021/ACS.CGD.8B01463/SUPPL_FILE/CG8B01463_SI_001.PDF.

(7) Glazer, A. M. The Classification of Tilted Octahedra in Perovskites. *urn:issn:0567-7408* **1972**, *28* (11), 3384–3392. https://doi.org/10.1107/S0567740872007976.

(8) Aleksandrov, K. S. The Sequences of Structural Phase Transitions in Perovskites. *Ferroelectrics* **1976**, *14* (1), 801–805. https://doi.org/10.1080/00150197608237799.

(9) Woodward, P. M. Octahedral Tilting in Perovskites. I. Geometrical Considerations. *urn:issn:0108-7681* **1997**, *53* (1), 32–43. https://doi.org/10.1107/S0108768196010713.

(10) Howard, C. J.; Stokes, H. T. Group-Theoretical Analysis of Octahedral Tilting in Perovskites. *urn:issn:0108-7681* **1998**, *54* (6), 782–789. https://doi.org/10.1107/S0108768198004200.

(11) Maculan, G.; Sheikh, A. D.; Abdelhady, A. L.; Saidaminov, M. I.; Haque, M. A.; Murali, B.; Alarousu, E.; Mohammed, O. F.; Wu, T.; Bakr, O. M. CH3NH3PbCl3 Single Crystals: Inverse Temperature Crystallization and Visible-Blind UV-Photodetector. *Journal of*

Physical Chemistry Letters **2015**, *6* (19), 3781–3786. https://doi.org/10.1021/ACS.JPCLETT.5B01666/ASSET/IMAGES/LARGE/JZ-2015-01666T_0006.JPEG.

(12) Hsu, H. P.; Li, L. C.; Shellaiah, M.; Sun, K. W. Structural, Photophysical, and Electronic Properties of CH3NH3PbCl3 Single Crystals. *Sci Rep* **2019**, *9* (1). https://doi.org/10.1038/s41598-019-49926-z.

(13) Saidaminov, M. I.; Abdelhady, A. L.; Murali, B.; Alarousu, E.; Burlakov, V. M.; Peng, W.; Dursun, I.; Wang, L.; He, Y.; MacUlan, G.; Goriely, A.; Wu, T.; Mohammed, O. F.; Bakr, O. M. High-Quality Bulk Hybrid Perovskite Single Crystals within Minutes by Inverse Temperature Crystallization. *Nature Communications 2015 6:1* **2015**, *6* (1), 1–6. https://doi.org/10.1038/ncomms8586.
